# Rictor maintains endothelial integrity under shear stress

**DOI:** 10.3389/fcell.2022.963866

**Published:** 2022-11-10

**Authors:** Hui Li, Wen-Ying Zhou, Yi-Xian Liu, Yi-Yuan Xia, Chun-Lei Xia, Dao-Rong Pan, Zheng Li, Yi Shi, Shao-Liang Chen, Jun-Xia Zhang

**Affiliations:** ^1^ Department of Cardiology, Nanjing First Hospital, Nanjing Medical University, Nanjing, China; ^2^ Department of Intensive Medicine, The Affiliated Jiangning Hospital of Nanjing Medical University, Nanjing, China

**Keywords:** rictor, endothelial integrity, low shear stress, vascular endothelial-cadherin, von Willebrand factor

## Abstract

**Background:** Endothelial injury induced by low shear stress (LSS) is an initiating factor in the pathogenesis of various cardiovascular diseases, including atherosclerosis, hypertension, and thrombotic diseases. Low shear stress activates the mammalian target of rapamycin complex 2 (mTORC2) signaling pathway. Rictor, the main constituent protein of mTORC2, is involved in vascular development. However, the impact of conditional Rictor ablation on endothelial homeostasis, especially on endothelial-specific markers, such as vascular endothelial-cadherin (VE-cadherin) and von Willebrand factor (VWF), under blood flow stimulation is unclear.

**Objective:** We aimed to investigate whether endothelial Rictor is involved in maintaining vascular endothelial integrity and the potential role of Rictor in atheroprone blood flow-mediated endothelial injury.

**Methods and results:** Immunofluorescence staining showed that endothelial Rictor was successfully knocked out in a mouse model. Scanning electron microscopy (EM) detection revealed disruption of the endothelial monolayer in the thoracic aorta of Rictor-deficient mice. Furthermore, scanning electron microscopy and transmission electron microscopy showed that Rictor deletion disrupted endothelial integrity and expanded cell junctions in the left common carotid artery region. *In vitro*, low shear stress disrupted actin filament polarity and the promoted the translocation of vascular endothelial-cadherin, the key component of adherens junctions (AJs) in human umbilical vein endothelial cells. After Rictor downregulation by small interfering RNA, the translocation of vascular endothelial-cadherin and stress fibers increased. Rictor knockdown inhibited low shear stress-induced von Willebrand factor upregulation, and downregulation of vascular endothelial-cadherin decreased low shear stress-induced von Willebrand factor expression. These results suggest that vascular endothelial-cadherin/von Willebrand factor is a possible mechanism mediated by Rictor in the pathological process of low shear stress-induced endothelial injury.

**Conclusion:** Rictor is a key protein that regulates endothelial integrity under vascular physiological homeostasis, and Rictor mediates low shear stress-induced endothelial injury by regulating adherens junctions and von Willebrand factor.

## Introduction

The mammalian target of rapamycin (mTOR) is involved in the regulation of various cellular functions and is closely related to the development of cardiovascular diseases (CVDs) (Cornu et al., 2013; Saxton and Sabatini, 2017). mTOR is a noncanonical serine/threonine protein kinase of the PI3K-related kinase family that can form two distinct multiprotein complexes, a Raptor containing rapamycin-sensitive mTOR complex 1 (mTORC1) and a Rictor containing rapamycin-insensitive mTOR complex 2 (mTORC2) (Kim et al., 2017). In addition, mTORC2 is involved in the regulation of cell survival, cytoskeleton remodeling, and cell migration (Sato et al., 2016). Activation of the mTORC2 signaling pathway has cardioprotective effects (Yano et al., 2014; Sciarretta et al., 2015). Rictor is the core component of mTORC2 (Sato et al., 2016). Functional studies discovered that blood pressure was increased in adipose-specific Rictor knockout mice (Drägert et al., 2015). Smooth muscle-specific Rictor-deficient mice develop spontaneous pulmonary hypertension (Tang et al., 2018). Through macrophage-specific Rictor deletion, early atherosclerosis (AS) can be inhibited (Babaev et al., 2018). Additionally, mice with endothelial Rictor knockout exhibit embryonic lethality (Aimi et al., 2015; Wang et al., 2015), and conditional knockout of endothelial Rictor significantly reduced fibroblast growth factor-induced neovascularization in mice (Aimi et al., 2015). However, research on the effects of Rictor on the structure or function of the endothelium is generally lacking.

Loss of endothelial integrity or endothelial dysfunction is thought to be an initiating factor in CVDs, such as (Davies, 2009) and hypertension (Joo Turoni et al., 2022). The structural integrity of the vascular endothelium depends on the cell junctions between endothelial cells (ECs). Adherens junctions (AJs) are the main type of cell junctions. Vascular endothelial-cadherin (VE-cadherin) is the main protein component of AJs. VE-cadherin expression levels and its localization on the cell membrane are important for maintaining endothelial barrier function (Barilli et al., 2008). The intracellular C-terminus of VE-cadherin connects actin filaments (F-actin) through cytoplasmic catenin, thereby anchoring the transmembrane to extracellular cadherin and the intracellular cytoskeleton, which maintains normal vascular permeability (Lampugnani et al., 1995; Taha et al., 2019).

Shear stress, the frictional force imposed by blood flow, is involved in the regulation of endothelial integrity. At the branches and bifurcations of blood vessels, ECs are stimulated by oscillatory shear stress or low shear stress (LSS). Here, endothelial homeostasis is unbalanced, and AS is prone to occur. Conversely, in the area of straight blood vessels, unidirectional blood flow is observed, and physiological shear stress (PSS) has a protective effect on the vascular endothelium (Davies, 2009). However, research on the role of Rictor in endothelial integrity and VE-cadherin expression is lacking under conditions of shear stress.

von Willebrand factor (VWF) is another critical marker for endothelial homeostasis (Morini et al., 2018). Rajkumar indicated that VWF negatively regulated the permeability of the blood‒brain barrier (Noubade et al., 2008). VWF is a large multidomain multimeric glycoprotein; its main function is to participate in physiological hemostasis and thrombosis (Xiang and Hwa, 2016; Yamazaki et al., 2021). The mTOR kinase inhibitor can inhibit thrombosis without affecting intracellular VWF secretion (Spater et al., 2018). However, the relationship between VWF and Rictor in endothelial injury has not been reported. Our previous studies confirmed that LSS activated the mTORC2 signaling pathway *in vitro* (Zhang et al., 2013; Zhang et al., 2014). However, during mechanotransduction, the specific biological function of vascular endothelial Rictor needs to be further elucidated.

In this study, we aimed to verify the effect of Rictor on endothelial integrity under physiological homeostasis and on VE-cadherin and VWF expression under LSS-induced vascular endothelial injury.

### Reagents

Primary antibodies against Rictor (2114s) and Na/K-ATPase (3010s) were purchased from Cell Signaling Technology. Primary antibodies against VE-cadherin (ab 91064) and VWF (ab 11713) were obtained from Abcam. A primary antibody against VE-cadherin (sc-9989) was purchased from Santa Cruz Biotechnology. The primary antibody against Rictor (BF0063) was purchased from Affinity. A VWF primary antibody (AF 301018) was purchased from AiFang Biological. A primary antibody against GAPDH (60,004–1) was purchased from Proteintech. Small interfering RNA (siRNA) targeting Rictor (8649s) was purchased from Cell Signaling Technology. Actin-Tracker Red-Rhodamine (C2207s) was obtained from Beyotime. ESL was purchased from Trans Gen Biotech (DW101-02).

### Cell culture

Human umbilical vein endothelial cells (HUVECs) were obtained from the Cellbank of the Chinese Academy of Sciences (Shanghai, China). Cells were cultured in ECM (ScienCell) supplemented with 5% fetal bovine serum (FBS), 1% ECGs, and 100 U/ml penicillin and streptomycin at 37°C in a 5% CO2 incubator.

### Application of low shear stress

The parallel flow chamber was manufactured by Shanghai Medical Instrument School (Shanghai, China) as previously described (Zhang et al., 2014). Low shear stress (LSS, 2 dyn/cm2) and physiological shear stress (PSS, 15 dyn/cm^2^) were applied for 1 h.

### Animal models

All experimental mice used in this study complied with the guidelines for the management and use of laboratory animals and were approved by the Laboratory Animal Welfare Ethics Committee of Nanjing Medical University. All mice were raised and bred in Nanjing Hospital Affiliated to Nanjing Medical University (Laboratory Animal Center, Nanjing First Hospital) and Model Animal Research Center of Nanjing University. All mice were on a C57BL/6 background and maintained under a 12:12 h light/dark cycle.

Floxed Rictor (Rictor^fl/fl^, B005657) crossed with Cre recombinase under the control of the CDH5 promoter (CDH5-CreERT2, T006897). After several generations of breeding, we generated mice with homologous Rictor deficiency named Rictor^fl/fl^-CDH5-CreERT2 (Rictor^iΔEC^) mice. Rictor^fl/fl^ littermates were used as controls. Age- and body weight-matched 11-week-old male mice were used.

Ligation of the left common carotid artery (LCA) was used to simulate low shear stress *in vivo*. Briefly, three of four branches of the LCA [external carotid artery (ECA), internal carotid artery (ICA) and occipital artery (OA)] were ligated, whereas the superior thyroid artery (STA) was left intact. The right common carotid artery (RCA) served as the control group.

### Immunofluorescence staining

HUVECs and tissue sections were washed thrice with ice-cold PBS, fixed with 4% paraformaldehyde for 15 min, and then permeabilized with 0.1% Triton X-100 for 15 min at room temperature. Subsequently, samples were blocked in 5% BSA. The samples were then incubated overnight with primary antibodies, including anti-VWF (1:1,000, ab 11,713), anti-VE-cadherin (1:100, sc-9989), and anti-Rictor (1:100, BF0063). Secondary fluorescent antibodies were added for 1.5 h. DAPI (YEASEN, China) was used for nuclear staining. Images were obtained through confocal microscopy (Zeiss LSM 510, Oberkochen, Germany).

### F-actin staining

HUVECs were fixed with 4% paraformaldehyde (PFA) followed by permeabilization with 0.1% Triton X-100 in PBS. F-actin was stained with Actin-Tracker Red-Rhodamine (1:40).

### *En face* staining

The RCA and LCA were isolated from male mice and fixed with 4% paraformaldehyde for 60 min. *En face* staining for VE-cadherin (Abcam, ab 91064), Rictor (Affinity, BF0063) and VWF (Abcam, ab 11713) was performed. Confocal immunofluorescence images were captured by confocal microscopy (Zeiss LSM 510, Oberkochen, Germany).

**Transmission electron microscopy (TEM) and scanning electron microscope (SEM) sample preparation**

*In vivo*, RCA and LCA from Rictor^fl/fl^ and Rictor^iΔEC^ mice were fixed by immersion in 2.5% glutaraldehyde at 4°C for 2 h.

### Immunoblotting analysis

The membrane proteins were extracted from cells by using Mem-PER™ Plus Reagents (Thermo Fisher Scientific, 89842). The total proteins were cleaved by RIPA buffer (P0013B, Beyotime) containing complete protease inhibitor cocktail tablets and phosphorylase inhibitor (PhosSTOP, Cat No. 04906845001, Roche, IN). Protein extracts were subjected to sodium dodecyl sulfate‒polyacrylamide gel electrophoresis (SDS–PAGE, Yeasen Biotech) and then transferred to polyvinylidene fluoride membranes (Millipore). Primary antibodies against VE-cadherin (1:200; sc-9989), Rictor (1:1,000; 2114s), VWF (1:500; AF 301018), and GAPDH (1:10,000; 60,008-1) were used. Proteins were visualized with HRP-conjugated anti-rabbit or anti-mouse IgG (1:10,000; Biosharp) using the ECL chemiluminescence system.

### Total RNA extraction and real-time PCR analysis

Total RNA was extracted from cells using RNA extraction kits (Triton X-100 9002-93-1, Bopa). RNA samples were reverse-transcribed with ChamQ universal SYBR qPCR Master Mix (Vazyme), and the ABI 7900HT Real-Time PCR System (Life Technologies) was used for real-time PCR. The results were normalized to the GAPDH level. The primer sequences for CDH5 are 5′-GTT​CAC​GCA​TCG​GTT​GTT​CAA-3′ and. 5′-CGC​TTC​CAC​CAC​GAT​CTC​ATA-3’. The primer sequences for GAPDH are. 5′- GGA​GCG​AGA​TCC​CTC​CAA​AAT-3′ and 5′- GGC​TGT​TGT​CAT​ACT​TCT​CAT​GG-3′.

### Small interfering RNA transfection

HUVECs were cultured on glass slides (30 × 50 mm) to 80% confluence and then transfected with siRNAs against Rictor and CDH5 using Lipofectamine 3,000 (Invitrogen, Carlsbad, CA, United States) according to the protocols recommended by the manufacturer. The transfection efficiency was detected by immunoblotting.

### Statistical analysis

Data are presented as the mean ± SD of each experiment performed in triplicate. Student’s t test and ANOVA after Tukey’s multiple comparisons were performed using GraphPad Prism (version 8.0.1; GraphPad Software, Inc.). *p* < 0.05 was considered to indicate a statistically significant difference.

## Results

### Construction of a mouse model with conditional rictor knockout in endothelial cells

**FIGURE 1:**
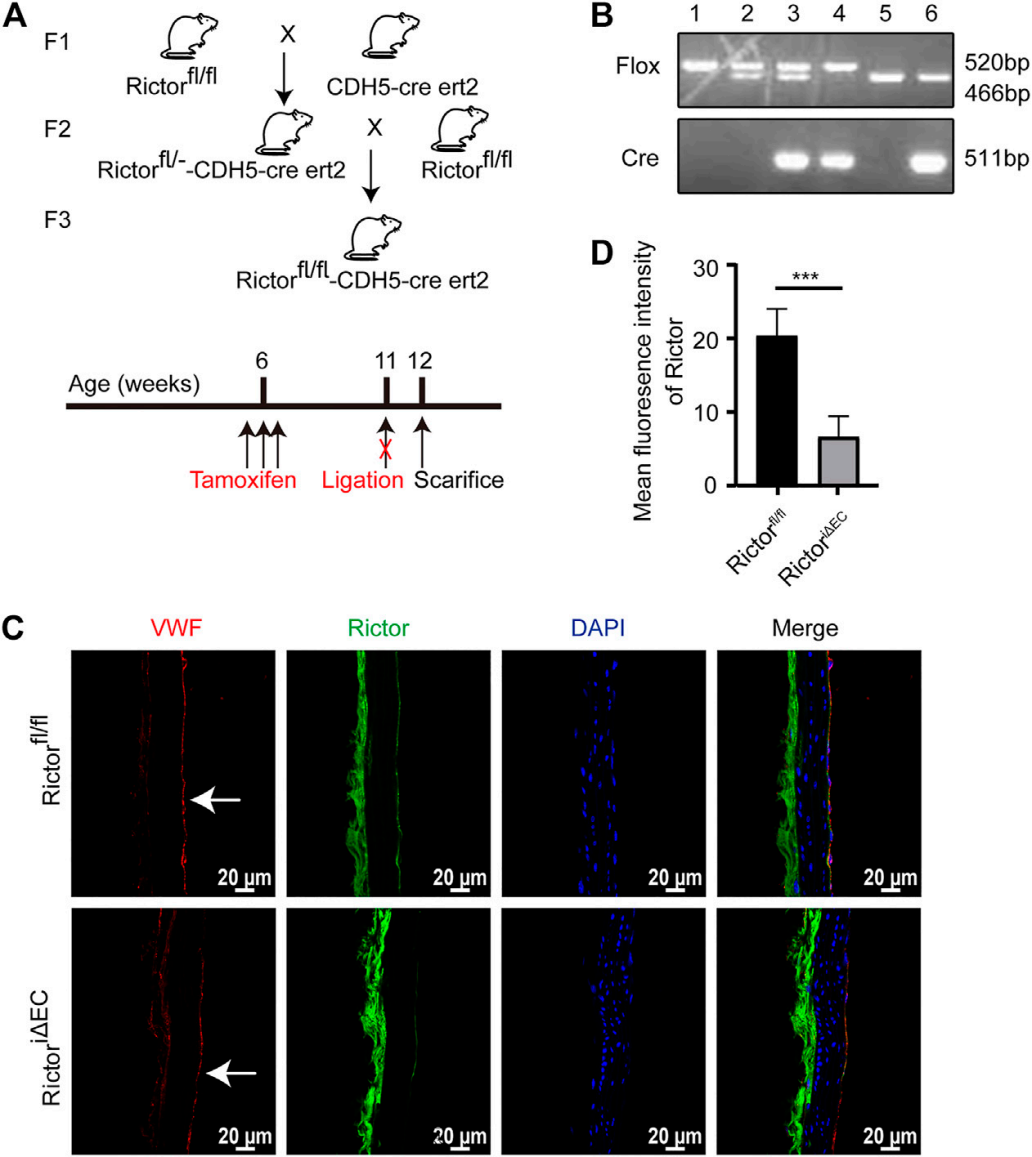
A mouse model with conditional Rictor knockout was constructed. **(A)** The breeding strategy for endothelial-conditional Rictor knockout mice is shown. **(B)** Partial mouse tail gene identification images are shown. 1: Rictor^fl/fl^, 2: Rictor^fl/-^, 3: Rictor^fl/—^CDH5-cre ert2, 4: Rictor^fl/fl^-CDH5-cre ert2, 5: WT, 6: WT-CDH5-cre ert2. **(C)** Representative images of Rictor expression in Rictor^fl/fl^ and Rictor^iΔEC^ (Rictor^fl/fl^-CDH5-cre ert2) mice are shown. Thoracic aortas were used for immunofluorescence here. And the white arrows indicate endothelial region. **(D)** The mean fluorescence intensity of Rictor is performed, scale bar = 20 μm, unpaired 2-tailed *t* test, n = 5, ***p = 0.0009 < 0.001.

To explore the effect of Rictor on the morphology of the vascular endothelium under physiological conditions, we first constructed a mouse model with time-controlled endothelium-specific deletion of Rictor. The breeding strategy was as follows (Figure 1A). Mice homozygous for LoxP-flanked Rictor (Rictor^fl/fl^) and mice expressing cre recombinase with the CDH5 promoter were crossed to obtain homozygous Rictor-deficient mice. The mice at 4–6 weeks of age were injected intraperitoneally with 50 mg/kg tamoxifen for five consecutive days. Mice with endothelium-specific ablation of Rictor were referred to as Rictor^iΔEC^ (Figure 1B, Lane 4), whereas age- and gender-matched Rictor^fl/fl^ littermates were considered controls (Figure 1B, Lane 1). Frozen sections of thoracic aorta were used for immunofluorescence (IF) staining. It showed that the expression of Rictor in endothelial layer was markedly reduced in Rictor^iΔEC^ mice compared with Rictor^fl/fl^ littermates (Figures 1C,D). The above results demonstrated that the endothelial conditional knockout Rictor mouse model was successfully established.

### Rictor deletion disrupted endothelial integrity in the laminar flow region

**FIGURE 2:**
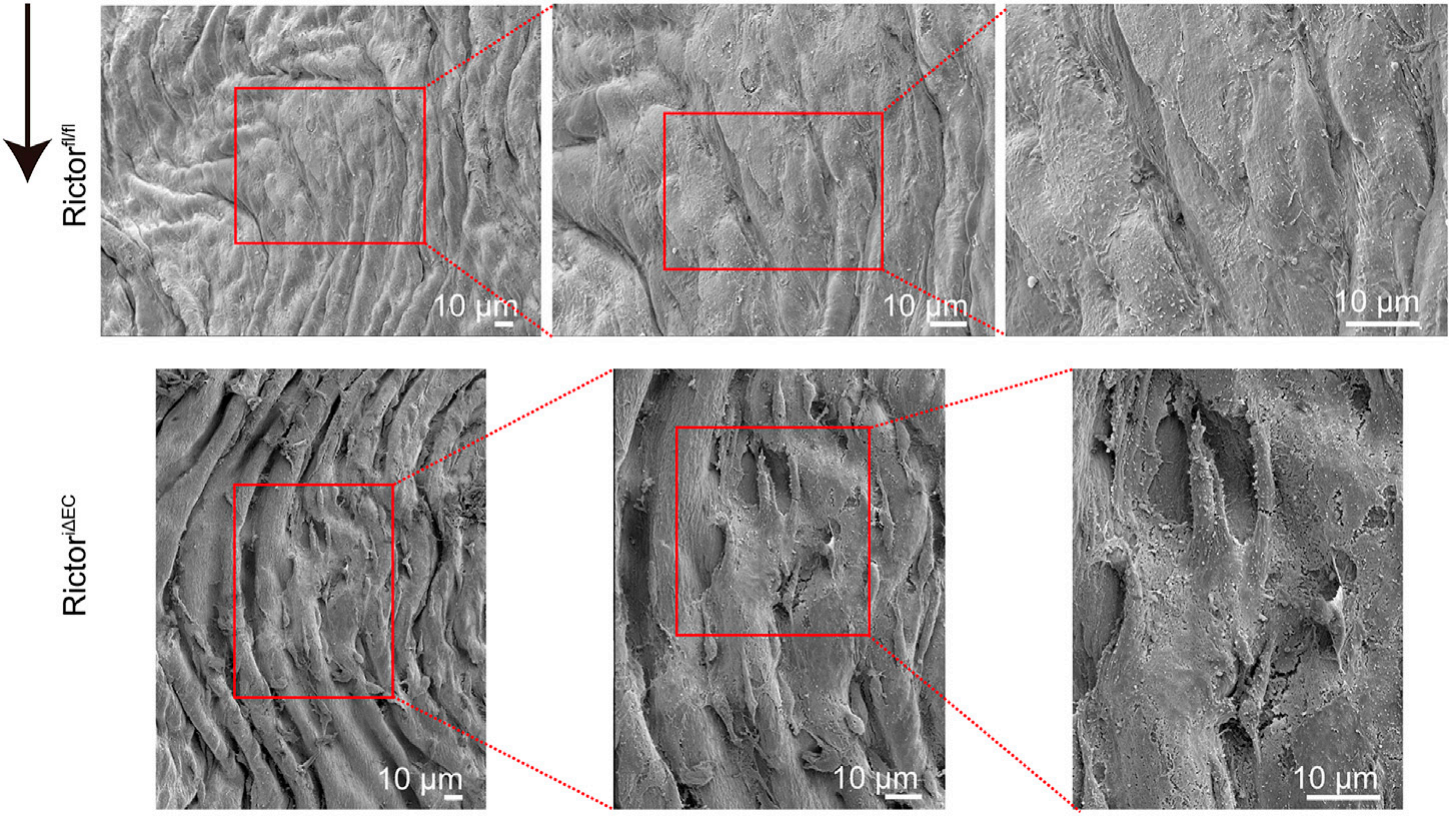
Rictor deletion disrupted endothelial integrity in the thoracic aortic region. Scanning electron microscopy images show the morphology of the thoracic aortic endothelium in Rictor^fl/fl^ (upper) and Rictor^iΔEC^ (lower) mice. The red boxed areas from left to right are local magnified areas, scale bar = 10 μm, n = 6. The black arrow indicates flow direction.

After successful generation of the mouse model of endothelial Rictor deletion, we selected the thoracic aortas of Rictor^fl/fl^ and Rictor^iΔEC^ mice for scanning electron microscopy (EM) to detect the endothelial morphology, where the endothelium is exposed to PSS. The results showed that endothelial integrity was damaged after Rictor deletion (Figure 2). The above results indicated that endothelial Rictor deletion can induce the loss of endothelial integrity.

### Rictor deletion disrupted endothelial integrity and expanded cell junctions in the left common carotid artery region

**FIGURE 3:**
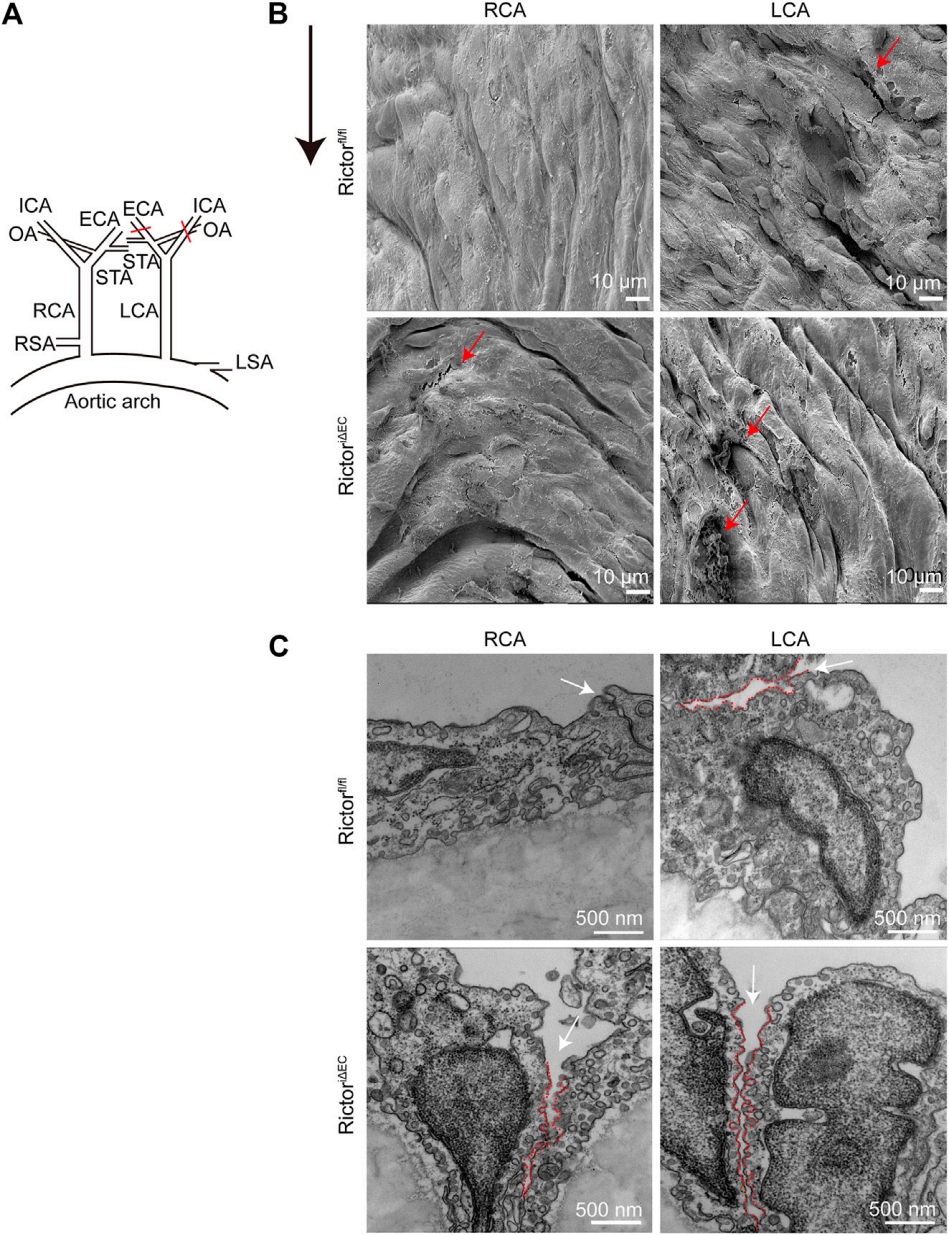
Effects of Rictor deletion on endothelial morphology under different types of shear stress. **(A)** Partial ligation of the LCA *in vivo* simulated LSS pattern, LCA: left common carotid artery, RCA: right common carotid artery, ECA: external carotid artery, ICA: internal carotid artery, OA: occipital artery, STA: superior thyroid artery, RSA: right subclavian artery, LSA: left subclavian artery. **(B)** Representative images of vascular morphology are observed by scanning electron microscopy. The red arrows indicate the damaged endothelium, scale bar = 10 μm, n = 6. The black arrow indicates flow direction. **(C)** Representative images of the connection between endothelial cells are observed by transmission electron microscopy. The white arrow indicates the widening of the gap at the junction between cells, and the red dotted line outlines the gap between cells, scale bar = 500 nm, n = 6.

To mimic LSS in a pathological state *in vivo*, we partially ligated the LCA in mice. The right common carotid artery (RCA) was not ligated in the sham control (Figure 3A). Scanning EM detection showed that the vascular endothelium of LCA was injured, and Rictor^iΔEC^ mice showed more pronounced endothelial morphological damage (Figure 3B). In addition, cell‒cell junctions were observed by transmission EM (Figure 3C). The cell junctions were widened by LSS stimulation, and the gaps in the cell junctions in the LCA of Rictor^iΔEC^ mice were more obvious. The above results showed that the absence of Rictor is involved in the pathological process of vascular endothelial injury caused by LSS.

### Rictor deletion promoted stress fibers formation

Next, we explored the mechanism by which Rictor deletion aggravated LSS-induced vascular endothelial morphological damage. F-actin is an important component of the endothelial cell junction complex. In the basal state, F-actin is located at the periphery of the cell membrane in complete and continuous forms and combines with the VE-cadherin complex to form linear AJs to maintain endothelial barrier function (Taha et al., 2019).

**FIGURE 4:**
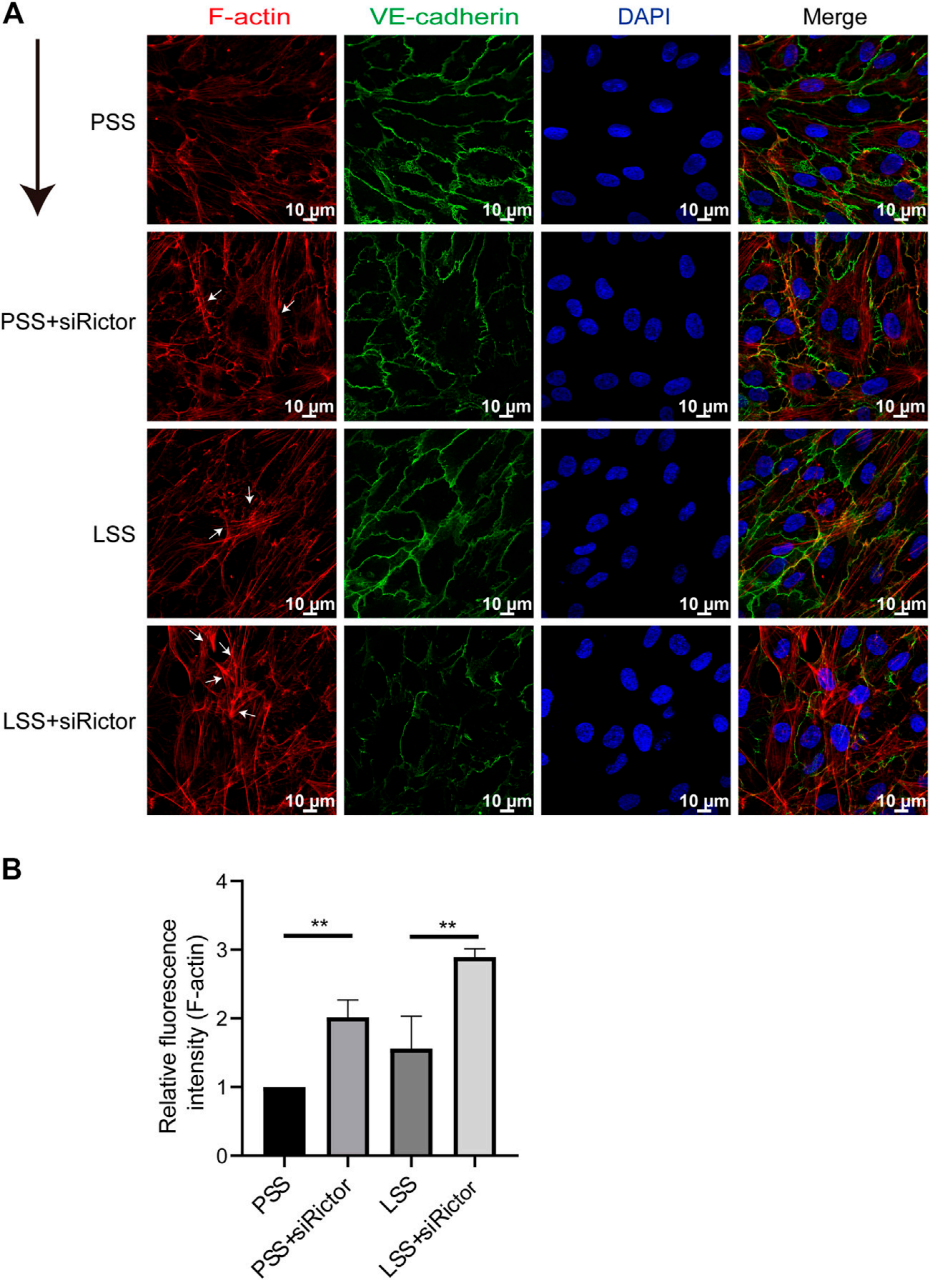
Rictor participated in the arrangement of the cytoskeleton under shear stress. **(A)** Immunofluorescence images show the polarity of actin filaments (F-actin) stimulated by LSS and with or without Rictor siRNA transfection. PSS and LSS represent physiological shear stress and low shear stress, respectively. The white arrows indicate the formation of stress fibers, scale bar = 10 μm. The black arrow indicates flow direction. **(B)** The mean fluorescence intensity of F-actin is measured, one-way ANOVA, n = 3, **p = 0.0084 < 0.01, **p = 0.0016 < 0.01.

*In vitro*, the stimulation of HUVECs with different types of shear stress was simulated in a parallel-plate flow chamber system. IF showed that F-actin was mainly distributed evenly around the cell under PSS conditions. After HUVECs were stimulated with LSS for 1 h, the polarity of F-actin was disturbed. Significant stress fibers formation was observed. And enchanced stress fibers formation was observed after silencing Rictor under LSS conditions (Figures 4A,B). This finding indicated that Rictor was important for the arrangement of cytoskeletal proteins, suggesting that Rictor may be involved in the morphological changes of ECs induced by LSS by regulating the distribution and arrangement of F-actin.

### Low shear stress induced adherens junctions disruption

**FIGURE 5:**
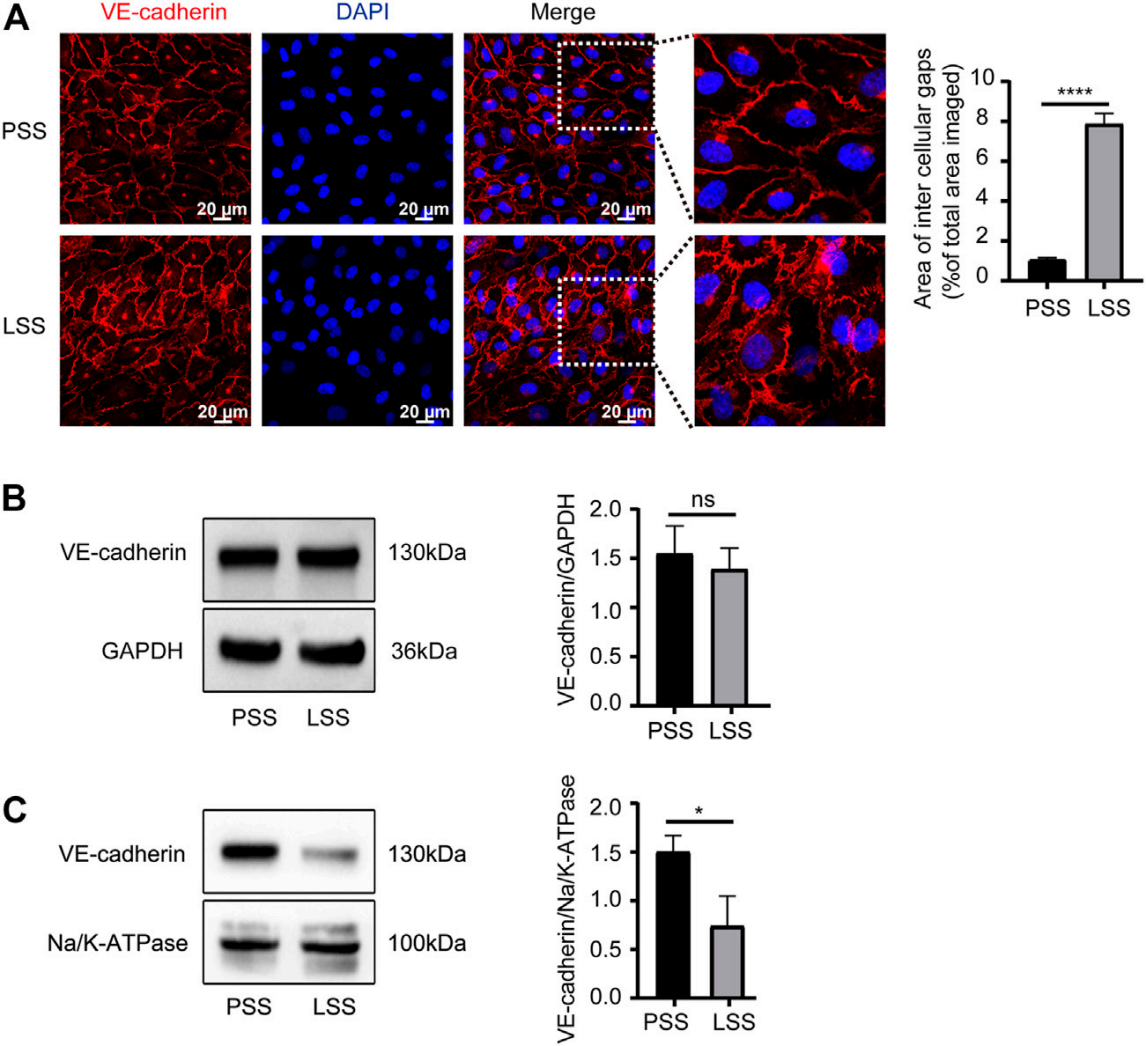
LSS promoted translocation of VE-cadherin. **(A)** Immunofluorescence shows the localization of VE-cadherin under different types of shear stress; scale bar = 20 μm. The magnified areas are on the right. VE-cadherin is less localized on the membrane after LSS stimulation. The right panel shows the quantification of the intercellular gaps, unpaired two-tailed *t* test, n = 3, ****p < 0.0001. **(B)** Total VE-cadherin protein expression is detected under different types of shear stress. The right panel shows the quantification, unpaired two-tailed *t* test, n = 3, ns indicates no statistical significance. **(C)** The expression of VE-cadherin on the cell membrane is detected under different types of shear stress. The right panel shows the quantification, unpaired two-tailed *t* test, n = 3, *p = 0.0195 < 0.05.

Endothelial AJs play a critical role in maintaining endothelial integrity. *In vitro*, the expression and localization of VE-cadherin, the main protein of AJs, were observed. IF demonstrated that VE-cadherin was mainly localized on the cell membrane under PSS stimulation with clear and smooth staining at cell junctions. However, under LSS stimulation, VE-cadherin was transferred into the cytoplasm partially with a decreased jagged shape at the cell junction. Additionally, obvious formation of intercellular gaps was noted (Figure 5A). Then, VE-cadherin protein expression in HUVECs was detected by immunoblotting. LSS caused decreased membrane protein expression of VE-cadherin (Figure 5C), whereas total protein expression was unchanged (Figure 5B). The above results indicated that the stability of VE-cadherin at AJs decreased during LSS exposure followed by translocation of VE-cadherin.

### Rictor downregulation reduced vascular endothelial-cadherin expression and its membrane localization under low shear stress stimulation

**FIGURE 6:**
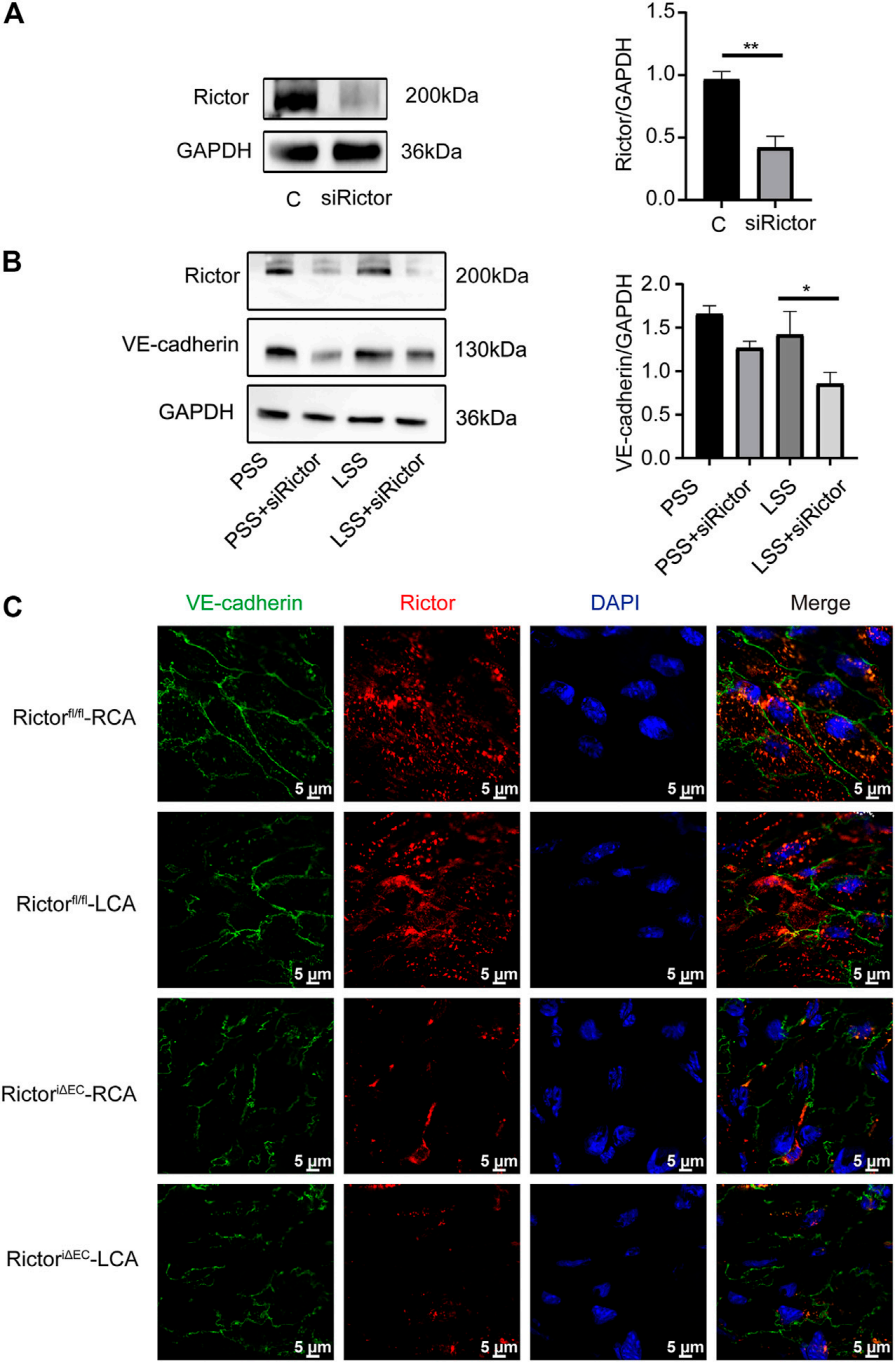
Rictor downregulation reduced VE-cadherin expression and its membrane localization under LSS stimulation. **(A)** Human umbilical vein endothelial cells transfected with Rictor siRNA. The right image is the quantification image, unpaired two-tailed *t* test, n = 3, **p = 0.001. **(B)** Representative images of VE-cadherin under different types of shear stress and with or without Rictor siRNA transfection are shown. The quantification data are presented on the right, one-way ANOVA, n = 3, *p = 0.0114 < 0.05. **(C)** *En face* staining showed the expression and localization of VE-cadherin under different shear stress and Rictor deletion conditions, scale bar = 5 μm.

Next, whether Rictor affected the integrity of ECs through VE-cadherin. *In vitro*, the transfection efficiency of Rictor siRNA was verified (Figure 6A). HUVECs were transfected with Rictor siRNA and then stimulated with PSS or LSS for 1 h. Immunoblotting showed that downregulation of Rictor suppressed VE-cadherin expression under LSS stimulation (Figure 6B). *In vivo*, *en face* staining showed that the total VE-cadherin and the localization of VE-cadherin on the cell membrane decreased in the LCA of Rictor^iΔEC^ mice (Figure 6C). The above results suggested that Rictor deletion aggravated the internalization of VE-cadherin and remodeling and F-actin in HUVECs stimulated by LSS, which was an important mechanism leading to endothelial morphological damage.

### Low shear stress promoted von Willebrand factor expression in endothelial cells

**FIGURE 7:**
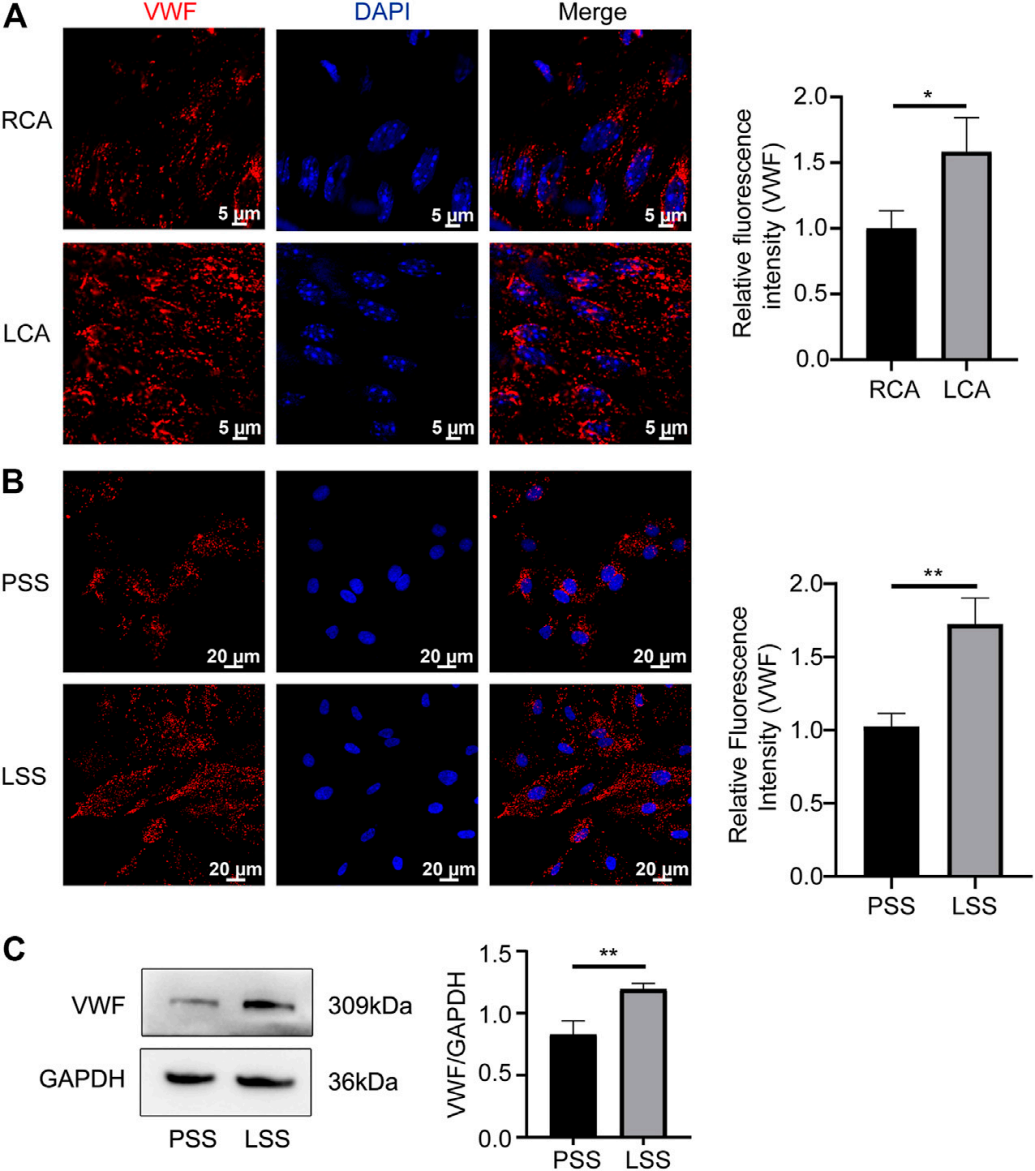
LSS promoted VWF expression. **(A)** Representative *en face* images of VWF, and the quantification image is shown on the right, unpaired two-tailed *t* test, n = 6, *p = 0.0262 < 0.05. **(B)** Representative immunofluorescence images of VWF under different types of shear stresses is shown unpaired two-tailed *t* test, n = 3, **p = 0.0036 < 0.01. **(C)** Representative immunoblotting images show that LSS promoted VWF expression. The quantification data for VWF are presented on the right, unpaired two-tailed *t* test, n = 3, 0.001 < **p = 0.0065 < 0.01.

VWF is another key protein that regulates vascular endothelial homeostasis, and its expression changes under different shear stress stimuli. *In vivo*, VWF was upregulated in the LCA compared to the RCA of Rictor^fl/fl^ mice (Figure 7A). *In vitro*, IF revealed that VWF was significantly upregulated after LSS stimulation (Figure 7B). Immunoblotting detection yielded consistent results (Figure 7C). The above data indicated that LSS upregulated VWF expression.

### Rictor downregulation suppressed low shear stress-induced von Willebrand factor expression

**FIGURE 8:**
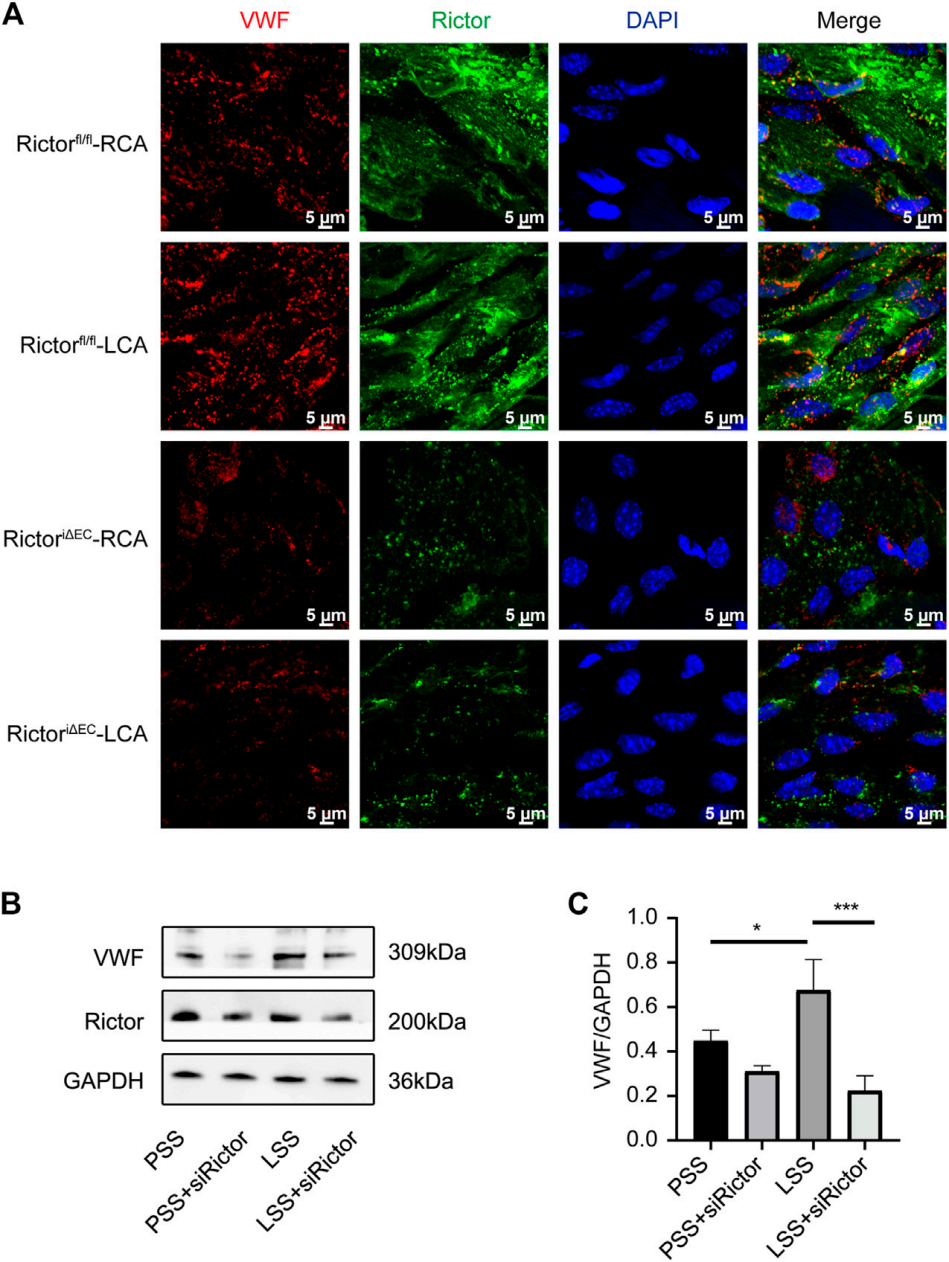
Downregulation of Rictor inhibited LSS-induced VWF expression. **(A)** *En face* staining shows representative images of VWF expression under different types of shear stress with Rictor presence or absence, scale bar = 5 μm. **(B)** Representative immunoblotting images show that downregulation of Rictor inhibited LSS-induced VWF expression. **(C)** Quantitative data for VWF normalized to GAPDH expression is show, one-way ANOVA, n = 3, *p = 0.0445 < 0.05, *p = 0.0294 < 0.05, ***p = 0.0005<0.001.

We next explored the role of Rictor in LSS-induced VWF expression. *En face* staining showed that Rictor downregulation suppressed LSS-induced VWF expression (Figure 8A). *In vitro*, siRNA was used to silence Rictor, and immunoblotting showed that Rictor downregulation inhibited LSS-induced expression of VWF protein (Figures 8B,C). The results implied that Rictor mediated VWF induced expression by LSS.

### Downregulation of vascular endothelial-cadherin reduced the low shear stress-promoted von Willebrand factor expression

**FIGURE 9:**
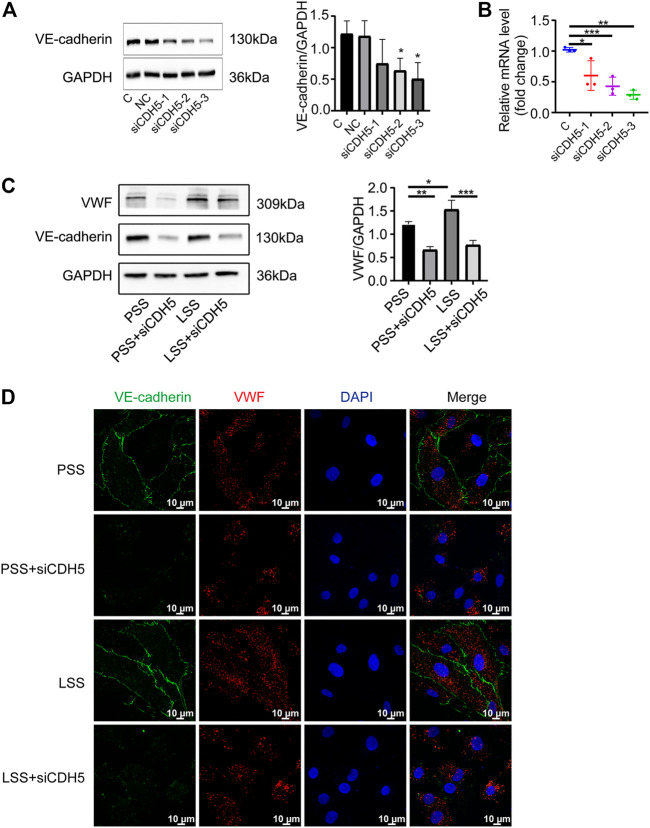
Downregulation of VE-cadherin reduced VWF expression. **(A)** Representative images show knockdown of VE-cadherin (CDH5). C and NC represent control and negative control group, respectively. The right image shows the quantification data, unpaired two-tailed *t* test, n = 3, *p = 0.0204 < 0.05, *p = 0.0239 < 0.05. **(B)** PCR detection of CDH5 knockdown efficiency is showed, unpaired two-tailed *t* test, n = 3, *p = 0.0410 < 0.05, ***p < 0.0001, **p = 0.0022 < 0.01. **(C)** The expression of VWF protein is detected by immunoblotting under different types of shear stress and with or without siCDH5 siRNA transfection. The right panel shows the quantitative data, one-way ANOVA, n = 3, *p = 0.0496 < 0.05, 0.0001 < ***p = 0.0002 < 0.001, **p = 0.0022 < 0.01. **(D)** Immunofluorescence images reveal that downregulation of VE-cadherin reduced VWF expression, scale bar = 20 μm.

To explore the effect of VE-cadherin on VWF expression under LSS stimulation, we first used immunoblotting (Figure 9A) and PCR (Figure 9B) detection to verify the three sequences of siRNA for CDH5 (the gene name of VE-cadherin) and selected the sequence with the best knockdown effect for subsequent experiments. Immunoblotting and IF detection showed that the upregulation of VWF expression induced by LSS stimulation was dependent on VE-cadherin (Figures 9C,D). These results indicated that LSS regulated VWF at least partly through VE-cadherin and suggested that the Rictor/VE-cadherin/VWF pathway mediated LSS-induced endothelial integrity disruption.

## Discussion

In our study, we demonstrate that Rictor plays a critical role in maintaining vascular endothelial integrity and participates in the mechanotransduction of shear stress. 1) Rictor is a key protein regulating endothelial integrity under physiological conditions. 2) Rictor deletion aggravated LSS-induced rearrangement and internalization of VE-cadherin and F-actin at AJs, suggesting that Rictor participated in LSS-induced endothelial morphological remodeling. 3) Rictor mediated LSS-induced VWF expression. 4) Rictor participates in mechanotransduction through the VE-cadherin/VWF pathway to regulate the homeostasis of vascular ECs.

The vascular endothelium is a monolayer of flattened cells lining the lumen surface of blood vessels. Endothelial damage is the initiating factor for many CVDs. The integrity of the vascular endothelium depends on close cellular connections and communications as well as the coordination of complex functions between cells and the extracellular matrix (ECM). Cell junctions refer to the cell‒cell connection structure, which includes gap junctions, tight junctions, and AJs (Garcia et al., 2018). In this study, we found ultrastructural alterations in cell junctions in conditional endothelial-Rictor ablation mice. Given that cell junctions build up a natural barrier against harmful circulating stimuli, our data provide pivotal evidence that Rictor may endow a “permissive” effect on endothelial integrity under physiological homeostasis.

Vascular ECs are directly stimulated by fluid shear stress, and identifying the mechanism of mechanotransduction is the key to understanding the effects of shear stress on cellular physiological processes, such as cell migration, regulation of cell morphology, and cell permeability (Sonmez et al., 2020). AJs are important mechanotransduction hubs. In this study, we found obvious changes in the subcellular localization of VE-cadherin in ECs after LSS stimulation for 1 h. Caolo and his colleagues found that LSS stimulated VE-cadheirn phosphorylation (Caolo et al., 2018). However, VE-cadherin expression was downregulated with the extension of LSS treatment time to 24 h (Seebach et al., 2007).

Relative to mTORC1, which has been extensively studied in the control of cell growth, proliferation, autophagy, and glycolipid metabolism (Jhanwar-Uniyal et al., 2019), relatively little research has been performed on mTORC2. A study showed that downregulation of Rictor impaired the recovery of hepatocyte growth factor secreted by mesenchymal stem cells to LPS-stimulated pulmonary microvascular endothelial barrier function (Meng et al., 2021). Downregulation of mTORC2 exacerbated the LPS-induced decrease in VE-cadherin expression in pulmonary microvascular ECs (Bieri et al., 2009). Rictor ablation in intestinal epithelial cells interrupts mTORC2/Akt signaling and increases epithelial permeability (Felipe et al., 2021). However, TORC2 activity is required for endocytosis and its inhibition results in reduced endocytosis (Margot et al., 2019). Our previous study revealed that LSS activated mTORC2 but not mTORC1 to induce endothelial oxidative stress (Zhang et al., 2014). However, the detailed role of Rictor under shear stress remains to be elucidated. We found that Rictor function normally maintains the expression of VE-cadherin and localization at AJs under LSS.

The cytoskeleton is a fibrous protein matrix mainly composed of microtubules, microfilaments, and intermediate filaments, of which microfilaments are mainly composed of three types of proteins (actin, myosin, and actin-binding proteins) (Shasby et al., 1982). F-actin is the main component of the cytoskeleton. In addition, the actin cytoskeleton is essential for the stabilization, remodeling, and mechanosensitive properties of AJs. The actin cytoskeleton acts as a dynamic push-pull system, where push forces maintain extracellular VE-cadherin interactions and pull forces stabilize intracellular adhesion complexes. In the basal state, F-actin is mainly distributed in the periphery of the cell, maintaining the normal shape of cells together with the interaction of adhesion molecules between cell‒cell and cell-ECM. In the context of stimulation, the cytoskeleton undergoes reorganization and redistribution. Numerous stress fibers develop in the center of the cell, resulting in increased central tension and mechanical imbalance at the AJs. Consequently, the gaps between cells increase, and vascular permeability increases (Liu et al., 2009; Efimova and Svitkina, 2018). We proposed that Rictor deletion promoted stress fibers under LSS stimulation.

VE-cadherin is involved in the regulation of VWF protein levels stimulated by LSS. Applying oscillatory shear stress to ECs for 6 h enhanced the exocytosis of VWF, triggering platelet aggregation and thrombosis (Zhu et al., 2020). Nevertheless, the permeability of the blood‒brain barrier in VWF knockout mice was increased, suggesting that VWF negatively regulates vascular endothelial permeability (Noubade et al., 2008). In ECs with thrombomodulin (TM)-specific deletion, thrombin resulted in the loss of VE-cadherin at the cell junction, decreased F-actin aggregation, and increased basal cell permeability and VWF expression. Knockdown of VWF in TM^−/−^ cells did not restore basal cell permeability, suggesting that the increase in VWF is independent of cell permeability (Giri et al., 2021). Interestingly, most previous studies have focused on the association of VWF secreted by Weibel-Palade bodies (WPBs) exocytosis with inflammation and thrombosis, whereas few studies have focused on VWF assembly and storage in WPBs. RNA-sequencing analysis indicated that VE-cadherin upregulated VWF through a transcriptional mechanism (Morini et al., 2018) because the VE-cadherin/β-catenin complex relieved the repression of the VWF gene promoter by the nuclear transcription of FoxO1. Previous studies suggest that VE-cadherin and VWF coordinate with each other to affect endothelial function homeostasis. This notion is consistent with our findings.

In summary, our study reported for the first time that Rictor plays a role in maintaining vascular endothelial integrity and mediating endothelial injury by regulating endothelial AJs and VWF under LSS. Additionally, Rictor affects the AJs and VWF protein under both PSS and LSS, suggesting that it may be a mechanosensor, and its existence plays an important role in the process of mechanotransduction.

## Data Availability

The original contributions presented in the study are included in the article/supplementary material, further inquiries can be directed to the corresponding authors.
